# Gelation and Crystallization Phenomena in Polyethylene Plastomers Modified with Waxes

**DOI:** 10.3390/polym13132147

**Published:** 2021-06-29

**Authors:** Markus Gahleitner, Jingbo Wang, Floran Prades, Klaus Bernreitner

**Affiliations:** Borealis Polyolefine GmbH, Innovation Headquarters, St. Peterstraße 25, 4021 Linz, Austria; Jingbo.Wang@borealisgroup.com (J.W.); floran.prades@borealisgroup.com (F.P.); klaus.bernreitner@borealisgroup.com (K.B.)

**Keywords:** polyethylene, plastomer, polypropylene, crystallization, rheology, transparency, mechanics

## Abstract

Polyethylene (PE) plastomers, single-site catalyst-based homogeneous linear low-density PEs (LLDPEs), combine low crystallinity, softness, and elasticity, making them ideal candidates for numerous applications such as hot-melt adhesives (HMA). As plastomers crystallize rather slowly, a number of possible low molecular weight polyolefin components were tested to accelerate solidification. An ideal modifier should accelerate solidification while maintaining transparency and softness of the base polymer. A Queo plastomer type was modified with different PE and PP waxes at concentrations of 5 to 25 wt.-%. Next to conventional calorimetry, a rheological technique was applied to study solidification. The resulting morphology was studied by atomic force microscopy, and the final compositions were investigated regarding their mechanical and optical performance. Accelerated solidification was observed in all cases, but a quite different course of structure formation could be concluded. PE waxes dissolve in the melt state, forming a lamellar network during cooling, whereas PP waxes form a heterogeneous blend in the melt for which the wax droplets solidify before the matrix. The particulate-type modification by the PP wax also affects stiffness less while retaining transparency better.

## 1. Introduction

Controlling the crystallization of polyolefins in order to facilitate or even enable processing at economical speed is a constant challenge in the polymer industry. The application of nucleating agents [1,2,3,4,5,6], but also of minor blend components [7,8], has been studied in the past more for isotactic polypropylene (iPP) than for polyethylene (PE), simply because the latter normally crystallizes much faster. This is quite different for PE plastomers, which are essentially single-site catalyst-based homogeneous linear low-density PEs (LLDPEs) combining low crystallinity, softness, and elasticity [9,10]. These advantages, however, go together with rather slow solidification and the generation of a micellar instead of the usual lamellar structure [11,12], which is elementary for the softness but at the same time limits processing speed. Recent work in the group of Prof. Schick in Rostock [13] performed at high cooling rates up to 1000 °C s^−1^ consequently showed that the increased crystallization times go together with an increased tendency for quench-induced density reduction. Although there is still no real vitrification as observed for isotactic polypropylene (iPP), the thus generated structures are metastable and likely to change over time through post-crystallization.

In practice, this may cause problems in both production—such as in the underwater pelletization of polymers, and processing—such as in film blowing or use in hot-melt adhesives (HMAs). Nucleation is one possibility to improve the situation, but its value is expected to be limited, as the problem lies not in the limited nucleation density (N) but in the crystal growth rate (G), both of which are required for fast crystallization [14,15]. Blends of PE plastomers with high-density PE (HDPE) are known to crystallize faster, similar to those of long-chain branched low-density PE (LDPE) [16,17]. However, a rather high concentration of HDPE, such as ≥20 wt.-%, is necessary to shift the crystallization temperature Tc from 40 to 110 °C, which will change the mechanical performance of the base polymer too much [16]. This problem is less relevant in the opposite case, when PE plastomers are used as impact modifiers for iPP, where the crystalline matrix stabilizes the situation.

The present study was, however, directed at the behavior of low-density plastomer solidification either in pure form during pellet formation after polymerization, or during use as a hot-melt adhesive (HMA) component. In both cases, next to the actual final solidification, the increase of viscosity and formation of a gel structure through crystallization is also relevant, making the selection of rheological techniques to study these effects logical. To accelerate the gelation and solidification of PE plastomer, various low molecular weight polyolefin components—both commercially available ones and self-produced ones—were tested. The target was to find a modifier that accelerates solidification while maintaining the transparency and softness of the base polymer.

In addition to conventional techniques like differential scanning calorimetry (DSC) or sophisticated ones like time-resolved X-ray scattering, rheological techniques may provide insight into polymer crystallization. Especially for complex systems, phase separation, nucleation, and gel formation may be studied to improve the understanding of the processing phenomena of semicrystalline polymers. Temperature sweeps in the linear viscoelastic domain have been established as a method to investigate gelation phenomena related to nucleation and crystallization behavior under quasi-quiescent conditions, as in the works of Winter’s working group [18,19,20,21]. 

When studying nucleation effects in polypropylene (PP) homopolymers and random copolymers by soluble nucleating agents [3] via this technique, the fibril and network formation before the actual PP crystallization can be observed under quasi-quiescent conditions, i.e., within the limits of linear viscoelastic response [22,23,24,25]. In this way, the excellent transparency effect of sorbitol and trisamide types [22,23,25], as well as the similar function of novel oxalamide types [24], could be explained. In addition, the two saturation levels normally observed for this class of nucleating agents [23,26] become logical: The first plateau at lower concentration corresponds to a concentration sufficient for fibril formation, whereas the second plateau indicates a network structure. The stability of such networks is, however, limited and the orientation of the fibrils will enhance flow-induced structure formation as well, similar to orientation effects resulting from anisotropic particulate nucleating agents. In general, it has been found that the dynamic temperature sweep method can also be applied to study the activity of particulate nucleating agents, both under quiescent [27] and shear-enhanced [28] conditions.

While not related to the present study, it should be mentioned that rheological studies of crystallization have been expanded into shear-induced effects as well [29]. Different combinations of step shear and dynamic oscillation have been shown to work for both iPP [30,31] and PE [32]. Both the absolute deformation and shear rate achievable this way are, however, rather limited, making the thus received results less comparable to practice than those from specific high-shear devices [33].

Similar to the work presented here, some papers discuss PE waxes mixed with conventional high molecular weight PE as a minority [34] or majority [35] component. There is only one paper for a low-density PE (in this case a high-pressure copolymer with vinyl acetate, EVA) modified with PP wax [36]. In none of these references were rheology and phase structure or even the mechanical performance of the final blends discussed. In this, the present study presents new findings with high relevance for the production and application of modified PE plastomers. We limited the scope to slow cooling processes, as the work of Schick did not imply sufficient effects at the observed changes in Tc [13].

## 2. Materials and Methods

The commercial PE plastomer type Queo 8085LA of Borealis AG, Austria, with a density of 883 kg/m³, an MFR (190 °C/2.16kg) of 85 g/10min, and a melting temperature (Tm) of 75 °C, was used as base polymer for all investigations. Queo plastomers are a range of PE copolymers made possible by combining metallocene catalyst technology with a solution polymerization process, using 1-octene as comonomer. 

Two PE waxes and two PP waxes each were applied for modification:

- EW1 is the commercial product Licowax PE190 from Clariant, Germany, an HDPE wax with a melt viscosity of 25 Pa s at 140 °C and a melting point T_M_ of 126 °C.
- EW2 is the commercial product Licolub H4 from Clariant, Germany, a paraffin wax with a melt viscosity of 8.10–3 Pa.s at 120 °C and a softening temperature (T_SOFT_) of 111 °C.
- PW1 is an ethylene-propylene random copolymer (RACO) wax, produced by peroxide-mediated visbreaking [37] from the commercial ethylene-propylene random copolymer grade RJ377MO of Borealis AG, Austria, with an ethylene (C2) content of 3.7 wt.-%, an MFR (230 °C/2.16 kg) of 500 g/10 min, and a T_M_ of 150 °C.
- PW2 is a PP terpolymer wax produced by peroxide-mediated visbreaking from the commercial ethylene-propylene-butene terpolymer grade TD109CF of Borealis AG, Austria, with an MFR (230 °C/2.16 kg) of 500 g/10 min and a T_M_ of 133 °C.

The waxes were added to a small-scale twin-screw extruder, type ThermoPRISM TSE16 of ThermoFisher Scientific, UK, to the base polymer in concentrations of 5–25 wt.-%. Melt mixing was performed at temperatures of 190–230 °C followed by solidifying the melt strand in a water bath and pelletizing the resulting compositions. The compositions and basic properties of the various compositions are listed in Table 1 for the PE waxes and Table 2 for the PP waxes.

Basic characterization for all compositions and some references included melt flow rate (MFR) measured according to ISO 1133 at 190 °C and 2.16 kg load and differential scanning calorimetry (DSC). Melting and crystallization behavior under quiescent conditions was determined by DSC with a TA Instruments Q2000 (TA Instruments, DE, USA) device according to ISO 11357/3 on 10 mg samples. Melting and crystallization temperatures were obtained in a heat/cool/heat cycle with a scan rate of 10 °C/min between 30 °C and 180 °C. The crystallization temperatures were taken as the peaks of the exotherms in the cooling step, and the melting temperatures as the peaks of the endotherms in the second heating step.

Based on dynamic mechanical melt testing in accordance with ISO 6271-1 and -6, a temperature scan in rheology originally developed to study network formation and nucleation of PP by soluble nucleating agents [22,23] was performed for all compositions. The test was done on compression molded samples under a nitrogen atmosphere using a 25 mm diameter plate and plate geometry in an Anton Paar MCR 502 rheometer (Anton Paar, Austria) equipped with a closed heating chamber. The sample was initially heated up to 220 °C and stabilized in terms of stress, followed by a temperature sweep from 200 °C down to 40 °C, until reaching a temperature at which the storage modulus G’ reached a level of 10^6^ Pa. A frequency of 1 rad/s was used in combination with a cooling rate of 2 °C per minute, determining the various transitions, as discussed in detail below.

To determine the thermo-mechanical performance of the compositions, the glass transition temperature T_G_, the storage modulus G’(23 °C), and the softening temperature T_SOFT_ were determined by dynamic mechanical analysis (DMA) according to ISO 6721-7 using a Rheometrics ARES instrument (formerly Rheometrics, now TA Instruments, DE, USA) with a heating/cooling chamber. The measurements are done in torsion mode on compression molded samples (40 × 10 × 1 mm^3^) between −100 °C and +150 °C until reaching a temperature at which the storage modulus G’ reached a level of 106 Pa with a heating rate of 2 °C/min and a frequency of 1 Hz.

In order to investigate the morphology, the solid compositions were investigated by atomic force microscopy (AFM) on cryo-cut surfaces, using the compression-molded plaques for DMA. An Asylum Research MFP 3D instrument (Oxford Instruments, Abingdon, UK) was used in dynamic (AC) mode using a standard silicon cantilever. No sample preparation beyond cryo-cutting was necessary, as the modulus difference between the different components of the compositions was found to be sufficient for imaging.

Further mechanical and optical data were determined on the compositions showing a promising acceleration of the solidification behavior by wax addition, as only those could be converted into injection-molded specimens without problems (compositions with the paraffin wax EW2 were too sticky for this). Haze was determined according to ASTM D1003-00 on 60 × 60 × 1 mm³ plaques injection molded in line with EN ISO 1873-2 using a melt temperature of 200 °C. A Byk Haze-Gard instrument (BYK-Gardner, Geretsried, Germany) was applied. The tensile modulus and the elongation at break were determined in accordance with ISO 527 on injection molded type 5A specimens at a test speed of 1 mm/min for the linear range and 5 mm/min up to break. A Zwick/Roell (Ulm, Germany) All round tester was used.

## 3. Results and Discussion

**Figure 1:**
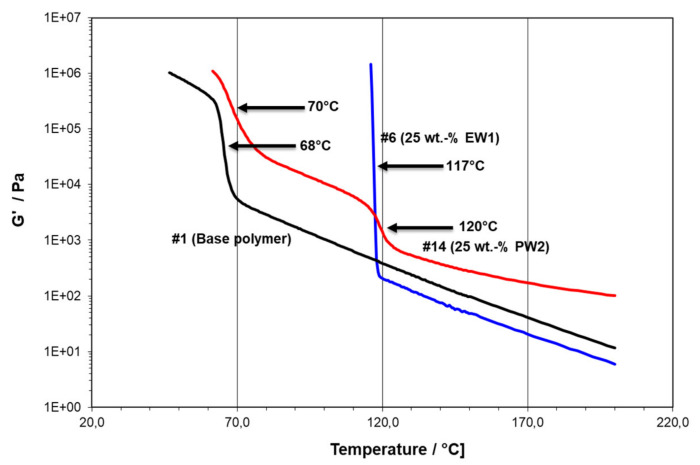
Temperature scans in cooling of materials #1, #6, and #14 (arrows indicating transition points taken for rheological transition temperatures).

At the core of the present study are the rheological temperature scans for determining possible network formations and crystallization effects. To demonstrate the possible courses of G’(T) during the cooling phase of these scans, Figure 1 compares the behavior of three compositions, illustrating the possible changes of storage modulus for the one- and two-component systems:

- -#1 is the pure PE plastomer type Queo 8085LA, with a clear solidification at 68 °C (T_C,RHEO_) and a measurable modulus in solid state;
- -#6 is modified with 25 wt.-% of PE wax EW1, with a gelation to a non-measurable modulus at 117 °C; and
- -#14 is modified with 25 wt.-% PP wax PW2, showing a first “network” transition at 120 °C (T_N,RHEO_) and a solidification at 70 °C (T_C,RHEO_).

In the case of double transitions, the higher one caused by the wax modifier was denominated “network transition” (T_N,RHEO_) in reference to earlier studies for nucleating agents [22,23], defined as the temperature at the inflexion point of this transition. The same was done for T_C,RHEO_ except for the cases where the rise was practically vertical, i.e., instantaneous. 

**Figure 2:**
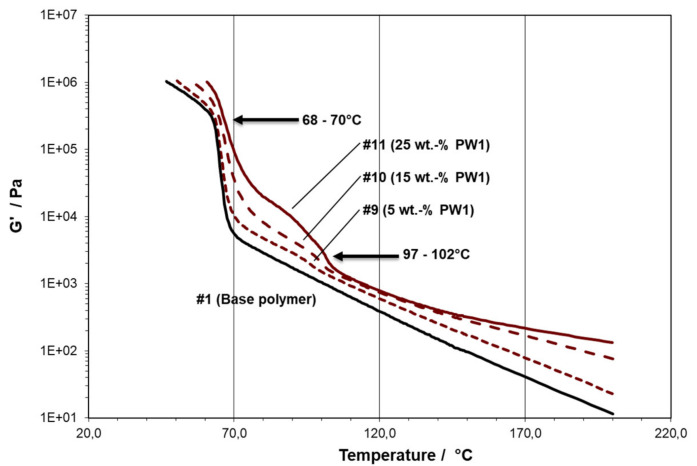
Temperature scans in cooling showing the concentration effect for PW1 (Material #1: 0 wt.-%, #9: 5 wt.-%, #10: 15 wt.-%, and #11: 25 wt.-%; arrows indicating transition points taken for rheological transition temperatures).

As can be seen from a comparison between Figure 2, presenting the concentration series for PP wax PW1, and Table 1 above, the “network” transition can also be identified for compositions not exhibiting a second T_C_ in DSC, meaning that the rheological method was more sensitive here. This double transition could only be observed for the modifications with PP wax, the concentration effects being very similar for PW1 and PW2.

**Figure 3:**
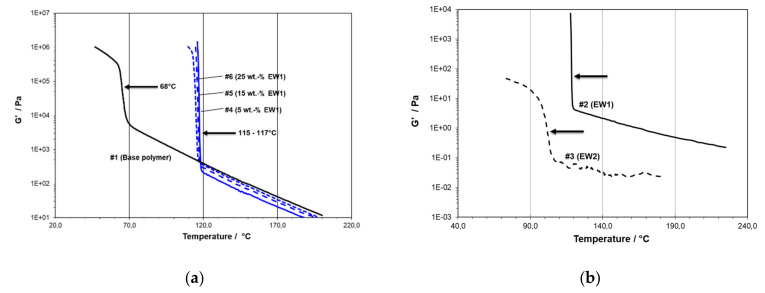
Temperature scans in cooling showing (**a**) the concentration effect for EW1 (Material #1: 0 wt.-%, #4: 5 wt.-%, #5: 15 wt.-%, and #6: 25 wt.-%) and (**b**) the behavior of pure modifier waxes EW1 (high molecular weight wax, #2) and EW2 (paraffin wax, #3; arrows indicating transition points taken for rheological transition temperatures).

Modification with PE wax EW1 gave a single transition at all levels, its T_C,RHEO_ only increasing slightly with concentration (see Figure 3a). The transition was also very rapid here and led to a G’-level above the measuring limit of 1 MPa, actually very similar to the behavior of the pure EW1 modifier (see Figure 3b). The much softer transition for the paraffin wax EW2 in the same diagram appeared to point to a solid-state modulus ~100 Pa at ambient temperature, which could not be measured directly as the material did not allow for the preparation of coherent specimens. Materials 7 and 8 modified with this wax type did not exhibit any effect beyond melt dilution above the solidification temperature of the base polymer.

In general, the melt part of the scans also showed whether the modifiers diluted the matrix (reducing viscosity, all PE waxes, see Figure 3) or thickened it (increasing viscosity, all PP waxes, see Figure 2). Next to the transition temperatures, the G’-level at 200 °C was also recorded as a measure for this. Together with the results from the DMA measurements discussed below, the mechanical and optical parameters, these data are summarized in Table 3 for PE and Table 4 for PP wax modification. 

**Figure 4:**
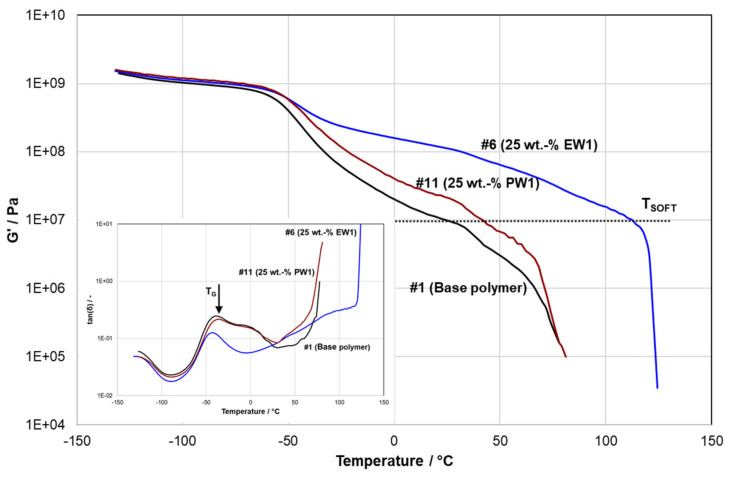
DMA temperature scans in heating of materials #1, #6, and #11 (main diagram for storage modulus, insert for loss angle).

Next to the requirement of accelerated solidification, the modification should not affect the solid-state properties or softening behavior of the modified plastomer unfavorably. DMA scans were used to check the effect at the glass transition temperature (T_G_), the stiffness at ambient temperature approximated by the storage modulus G’ at 23 °C, and the softening behavior. The softening temperature, T_SOFT_, was defined as the temperature where G’ falls below the level of 10 MPa. Figure 4 exemplifies the behavior of the base polymer and two modified compositions in reference to Figure 1, showing the significant stiffness increase by the PE wax EW1 and equally the change in softening behavior. In contrast, PP wax PW1, despite its higher melting point according to DSC (see Table 2), affected both significantly less. As the data summarized in Table 3 and Table 4 show, these effects were similar for both PP waxes, whereas the modification effect by the lower molecular weight paraffin wax EW2 was weaker than for EW1.

**Figure 5:**
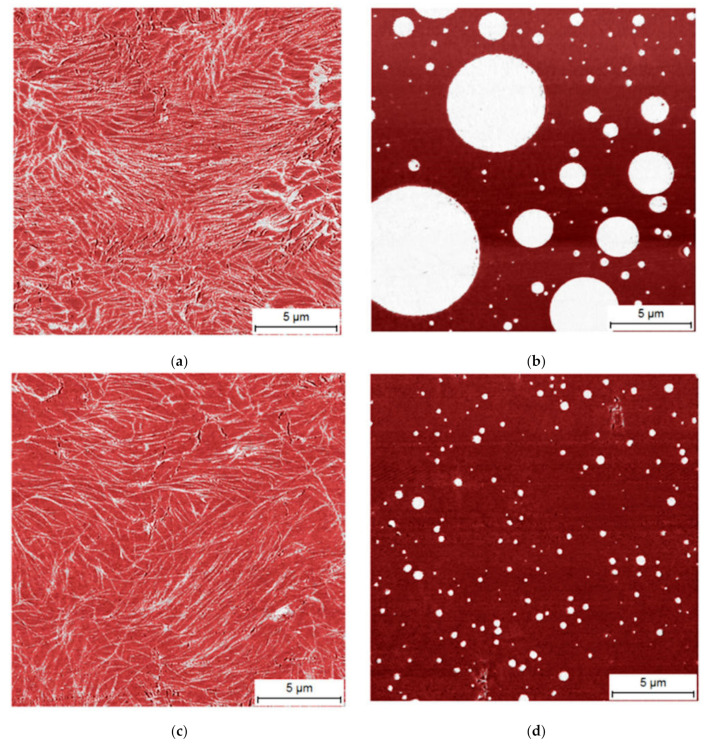
Phase morphology from AFM for (**a**) material #6 with 25 wt.-% EW1, (**b**) material #14 with 25 wt.-% PW2, (**c**) material #4 with 5 wt.-% EW1, and (**d**) material #9 with 5 wt.-% PW1.

In order to understand these different effects, the phase morphology of some of the modified materials was studied by AFM. In Figure 5a,b the strongest modified compositions with EW1 and PW2 are compared, basically explaining the difference between PE and PP wax effects. The dark background in both structures is the plastomer recorded with more loss component in tapping mode AFM, whereas the bright elements represent the more crystalline wax phases. For EW1, a network structure of lamellar crystals is observed, whereas the PW2 forms discrete and spherical particles.

At lower concentrations, the structural principle remained the same, and there was also no relevant difference between PW2 and PW1, as can be seen from Figure 5c,d. Together with the rheological data, this allows for the following conclusions: The higher molecular weight PE wax EW1 dissolved in the plastomer upon melting, lowering its viscosity significantly, but re-crystallized in lamellar form during cooling. The resulting lamellae formed a network sufficient for gelation in similar structures, as for HDPE crystallization from solutions [38]. For the paraffin wax EW2, the molecular weight was obviously too low to form stable lamellae, resulting in a much weaker effect.

In contrast to that, both PP waxes were found to be distributed as micro-droplets in the melt state still, due to their incompatibility with the plastomer, as also observed for conventional blends [39,40]. The concentration dependence of droplet size obviously resulted from mixing problems due to the viscosity differences. These droplets solidified well above the matrix crystallization temperature, causing a viscosity increase resembling a weak gel (T_N,RHEO_) proportional to their concentration. Likewise, in melting the PE wax network remained stable up to its melting point, whereas the still solid PP wax particles only increased the viscosity of the molten plastomer matrix.

**Figure 6:**
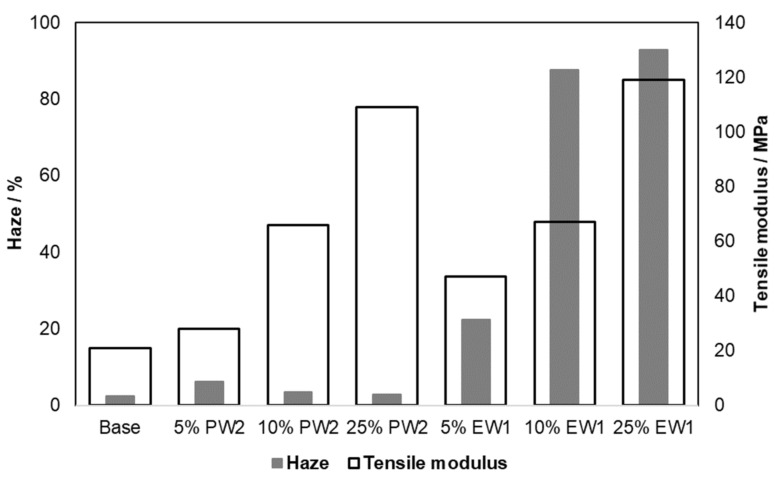
Haze (2 mm, ASTM D1003-00) and modulus (tensile, ISO 527) effects of PP wax PW2 and PE wax EW1.

To finally check practical application effects, the haze and tensile behavior for two of the concentration series—with EW1 and PW2—were tested on injection-molded specimens. As Figure 6 shows, the particulate-type modification by the PP wax not only affected stiffness resp. modulus less, but also retained the initial transparency of the PE plastomer much better. Considering both together with the accelerated solidification, the PP waxes as modifiers for hot-melt adhesive application were covered by a patent application [41].

## 4. Conclusions

For the development of PE plastomers and hot-melt adhesives (HMA) based on these, a number of possible low molecular weight polyolefin components were tested to accelerate plastomer solidification. The target was to find a modifier that accelerates solidification while maintaining the transparency and softness of the base polymer. The Queo 8085LA plastomer type was modified with different PE and PP waxes at concentrations of 5 to 25 wt.-%. Next to conventional differential scanning calorimetry (DSC), a rheological technique was applied to study solidification. The resulting morphology was studied by atomic force microscopy, and the final compositions were investigated regarding their mechanical and optical performance.

Accelerated solidification was observed in all cases, with a two-stage process of network formation followed by full gelation for the PP waxes and the paraffin wax, or in a one-stage process for the high molecular weight PE wax EW1. Together with DMA and AFM morphology studies, a quite different course of structure formation could be concluded: dissolution in the melt state and lamellar network formation in case of EW1, and a heterogeneous blend with PP wax droplets solidifying before the matrix for PW1 and PW2.

Both G’ from DMA and the tensile modulus further showed the particulate-type modification by the PP wax to affect stiffness, while also retaining the transparency better. Further optimization by improving the compatibility and viscosity ratio certainly seems possible.

## Figures and Tables

**Table 1:** Compositions and references for PE wax modification with MFR and DSC data (* ≥5000 g/10 min).

Composition		1	2	3	4	5	6	7	8
Queo 8085L	Wt.-%	100	0	0	95	85	75	95	75
EW1	Wt.-%	0	100	0	5	15	25	0	0
EW2	Wt.-%	0	0	100	0	0	0	5	25
MFR 190 °C	g/10 min	85	n.d. *	n.d. *	98	109	133	110	205
**DSC**									
T_C,1_	°C	-	114	97	109	110	113	60	62
T_C,2_	°C	56	-	-	61	61	60	-	85
T_M,1_	°C	54	-	-	72	74	73	62	62
T_M,2_	°C	76	126	109	121	124	79	78	86

**Table 2:** Compositions and references for PP wax modification with MFR and DSC data (see text above for data of PW1 and PW2).

Composition		1	9	10	11	12	13	14
Queo 8085L	Wt.-%	100	95	85	75	95	85	75
PW1	Wt.-%	0	5	15	25	0	0	0
PW2	Wt.-%	0	0	0	0	5	15	25
MFR 190 °C	g/10 min	85	83	79	78	84	82	80
**DSC**								
T_C,1_	°C	-	-	-	109	-	-	96
T_C,2_	°C	56	56	58	57	56	56	58
T_M,1_	°C	54	75	75	75	75	74	75
T_M,2_	°C	76	147	147	144	128	132	131

**Table 3:** Compositions and references for PE wax modification with rheology and DMA results.

Composition		1	2	3	4	5	6	7	8
Queo 8085L	Wt.-%	100	0	0	95	85	75	95	75
EW1	Wt.-%	0	100	0	5	15	25	0	0
EW2	Wt.-%	0	0	100	0	0	0	5	25
**Rheology**									
G’(200 °C)	Pa	12.0	0.60	0.02	9.2	8.0	5.9	8.4	1.2
T_N,RHEO_	°C	-	-	-	-	-	-	96	107
T_C,RHEO_	°C	67	120	102	115	116	117	69	70
**DMA**	°C								
T_G_	°C	–40	n.d.	n.d.	–41	–42	–43	–42	–42
G’(23 °C)	Pa	10	n.d.	n.d.	50	76	116	19	61
T_SOFT_	°C	24	n.d.	n.d.	71	85	112	38	57

**Table 4:** Compositions and references for PP-wax modification with rheology and DMA results.

Composition		1	9	10	11	12	13	14
Queo 8085L	Wt.-%	100	95	85	75	95	85	75
PW1	Wt.-%	0	5	15	25	0	0	0
PW2	Wt.-%	0	0	0	0	5	15	25
**Rheology**								
G’(200 °C)	Pa	120	22	69	100	23	76	132
T_N,RHEO_	°C	-	97	100	102	115	117	120
T_C,RHEO_	°C	67	68	69	70	68	69	70
**DMA**								
T_G_	°C	–40	–39	–39	–38	–39	–38	–38
G’(23 °C)	Pa	10	13	18	24	13	30	22
T_SOFT_	°C	24	132	39	42	33	37	41

## Data Availability

Additional data sufficient for reproducing the work as well as further mechanical data can be found in reference [41].
